# Davinci the Dualist: The Mind–Body Divide in Large Language Models and in Human Learners

**DOI:** 10.1162/opmi_a_00120

**Published:** 2024-03-01

**Authors:** Iris Berent, Alexzander Sansiveri

**Affiliations:** Department of Psychology, Northeastern University, Boston, MA, USA

**Keywords:** Dualism, learnability, AI, GPT, Davinci

## Abstract

A large literature suggests that people are intuitive Dualists—they consider the mind ethereal, distinct from the body. Furthermore, Dualism emerges, in part, via learning (e.g., Barlev & Shtulman, 2021). Human learners, however, are also endowed with innate systems of core knowledge, and recent results suggest that core knowledge begets Dualism (Berent, 2023a; Berent et al., 2022). The resulting question, then, is whether the acquisition of Dualism requires core knowledge, or whether Dualism is learnable from experience alone, via domain-general mechanism. Since human learners are equipped with both systems, the evidence from humans cannot decide this question. Accordingly, here, we probe for a mind–body divide in Davinci—a large language model (LLM) that is devoid of core knowledge. We show that Davinci still leans towards Dualism, and that this bias increases systematically with the learner’s inductive potential. Thus, davinci (which forms part of the GPT-3 suite) exhibits mild Dualist tendencies, whereas its descendent, text-davinci-003 (a GPT-3.5 model), shows a stronger bias. It selectively considers thoughts (epistemic states) as disembodied—as unlikely to show up in the body (in the brain). Unlike humans, GPT 3.5 categorically rejected the persistence of the psyche after death. Still, when probed about life, GPT 3.5 showed robust Dualist tendencies. These results demonstrate that the mind–body divide is partly learnable from experience. While results from LLMs cannot fully determine how humans acquire Dualism, they do place a higher burden of proof on nativist theories that trace Dualism to innate core cognition (Berent, 2023a; Berent et al., 2022).

## INTRODUCTION

A large literature suggests that people are intuitive Dualists—they view the mind as ethereal, distinct from the body (e.g., Bloom, 2004). Much of the evidence for Dualism, however, obtains from Western culture, where the dominant Judeo-Christian tradition explicitly endorses Dualism. The strong links between this explicit doctrine and intuitive Dualist beliefs raise the possibility that intuitive Dualism emerges solely via cultural transmission, by learning from experience (e.g., Hodge, 2008; Watson-Jones et al., 2017).

In line with this proposal, past research has indeed shown that intuitive Dualist beliefs differ across cultures (Astuti & Harris, 2008; Barrett et al., 2021; Cohen & Barrett, 2011; Harris & Giménez, 2005; Lane et al., 2016; Watson-Jones et al., 2017), and they change along development (e.g., Harris & Giménez, 2005; Shtulman, 2008; for review: Barlev & Shtulman, 2021). Still, whether learning alone is ***sufficient*** to give rise to intuitive Dualism is uncertain for several reasons.

First, intuitive Dualism is not solely a Western phenomenon. Evidence for Dualism has been observed across cultures (Boyer, 2018; Chudek et al., 2018; Cohen et al., 2011; Weisman et al., 2021), including in small scale societies (e.g., Chudek et al., 2018). The hypothesis that Dualism arises solely by learning fails to explain why Dualism is pervasive, cross-culturally.

Second, intuitive Dualism not only coarsely segregates mind from body (in line with the religious doctrine). Rather, people (adults and children) show granular distinctions that render thoughts (*epistemic states*) more ethereal than emotions, sensations and actions (hereafter, “*non-epistemic*” states; Berent, 2021, 2023b, 2023c; Berent & Platt, 2021; Berent et al., 2021, 2022; Bering & Bjorklund, 2004; Sandoboe & Berent, 2021). While the Western canon, no doubt, presents learners with much evidence for a coarse mind–body contrast, it is uncertain whether it can explain these granular distinctions. Thus, whether the experience available to Western learners is sufficient to induce the full set of intuitive Dualist beliefs is unknown.

Finally, and most significantly, humans are endowed not only with learning mechanisms but also with innate systems of core knowledge. Past research has suggested that intuitive Dualism can arise from innate core knowledge; specifically, from the tension between intuitive physics, on the one hand, and theory of mind, on the other (Bloom, 2004). In line with this proposal, recent studies have shown that individuals whose mind-reading abilities are weaker exhibit weaker intuitive Dualism; this is the case in autistic- relative to neurotypical individuals (Berent et al., 2022), and in neurotypical males relative to females (Berent, 2023c). It is the rooting of Dualism in core knowledge that presents the strongest evidence for its innate origins.

Accordingly, intuitive Dualism in humans could arise ***either*** because (a) Dualism is fully learned from experience, or because (b) Dualism emerges, in part, thanks to innate core knowledge. In this latter hypothesis (b), learning can contribute to the emergence of intuitive Dualism, but it may not be *sufficient* for it to emerge.

In light of these challenges, it is unclear whether Dualism arises solely by learning from experience. The existing results from humans do not settle this issue, as humans are not only prodigious learners but they are also arguably endowed with innate systems of core knowledge (Spelke, 1994) that could canalize the acquisition of Dualist intuitions. Thus, the evidence that humans *can* learn some of their Dualist intuitions from experience does not decide the question of whether intuitive Dualism is in principle ***learnable*** from experience alone.

To evaluate the role of learning, here, we gauge Dualism in Davinci—a large language model, created by OpenAI. Although Davinci’s performance remains imperfect (e.g., Binz & Schulz, 2023; Mitchell, 2023; Sap et al., 2022), it demonstrably approximates human learning in several domains (e.g., Dillion et al., 2023; Grossmann et al., 2023; but cf. Park et al., 2024); some have even credited it with general intelligence (Bubeck et al., 2023; but cf. Marcus, 2022a).

Unlike humans, however, Davinci is devoid of innate core knowledge (Marcus, 2022b). Thus, if the evidence available to humans is *sufficient* to induce a Dualist bias from experience, and Davinci adequately models human learning, then a Dualist bias ought to emerge in Davinci.

To reiterate, the evidence from Davinci cannot settle how humans effectively ***learn*** their Dualist bias, as humans and AI differ not only on the experience available to them but potentially, also on their learning mechanisms. Instead, our goal is to address the logically prior question of whether Dualism is, *in principle*, ***learnable***, in the absence of innate core knowledge.

This concern with learnability follows a long research tradition in language acquisition (Gold, 1967; Perfors et al., 2011; Pinker, 1984; Tesar & Smolensky, 1998). Below, we explore the contrast between what humans learn and what is learnable, and how the results from Davinci shed light on these questions.

## THE LEARNABILITY OF DUALISM

A large body of research has used evidence from artificial learners to probe the origins of human knowledge. The logic guiding this research program is simple: if the relevant knowledge is innate in humans, then that knowledge may not be learnable by artificial learners; success would thus challenge nativist accounts of cognition.

Following this line of reasoning, recent research has used the LLMs to evaluate nativist accounts of putatively innate core knowledge systems, such as language (Contreras Kallens et al., 2023; Piantadosi, 2024) and theory of mind (Bubeck et al., 2023; Kim et al., 2023; Kosinski, 2023; Sap et al., 2022; Shapira et al., 2023; Trott et al., 2023).

Here, we adopt a similar approach to explore the origins of Dualism. At first blush, this enterprise may seem puzzling. Unlike theory of mind and universal grammar, Dualism isn’t considered innate even by staunch nativists; indeed, Dualism offers no discernable selective advance. As such, it is quite likely that Dualism arises in humans partly by learning. And if learning mediates the acquisition of Dualism, then it would seem that innate knowledge plays little role in its acquisition. Thus, Dualism ought to be fully *learnable* from experience solely via domain-general learning mechanisms, such as LLMs.

We, however, believe that the learnability of Dualism by LLM is far from evident on both principled and empirical grounds. As a matter of principle, the possibility that learning likely informs the acquisition of Dualism does not, in and of itself, show that learning is *sufficient* for Dualism to emerge. And indeed, many species-specific behaviors arise by the conjunction of innate constraints and some triggering experience; birdsong is a quintessential example (e.g., Fehér et al., 2009). Thus, the fact that humans (who are likely equipped with innate core knowledge) acquire Dualism partly by learning does not prove that Dualism is fully learnable by LLMs (endowed with domain-general learning mechanisms only).

In addition to this principled problem, there are also empirical challenges to the learnability of Dualism. As noted, human Dualist intuitions are far more nuanced than the coarse mind–body contrast that is plainly patent in Western culture (e.g., religion). Moreover, evidence for Dualism emerges across human cultures. Whether learning alone can explain the widespread score of Dualism and its fine-grained contents is uncertain.

LLMs can shed light on the principled *learnability* of Dualism. As noted, the conclusions from LLMs do not directly speak to how humans acquire Dualism (as LLMs and humans are different learners, presented with vastly different input). Nonetheless, they can limit the range options.

To see how, let’s consider the intersection between two questions: (a) Is Dualism learnable, in principle; and (b) how human learners acquire Dualism. Our goal here is not to select the right answer but rather to detail the range of logical possibilities.

**Figure 1.**
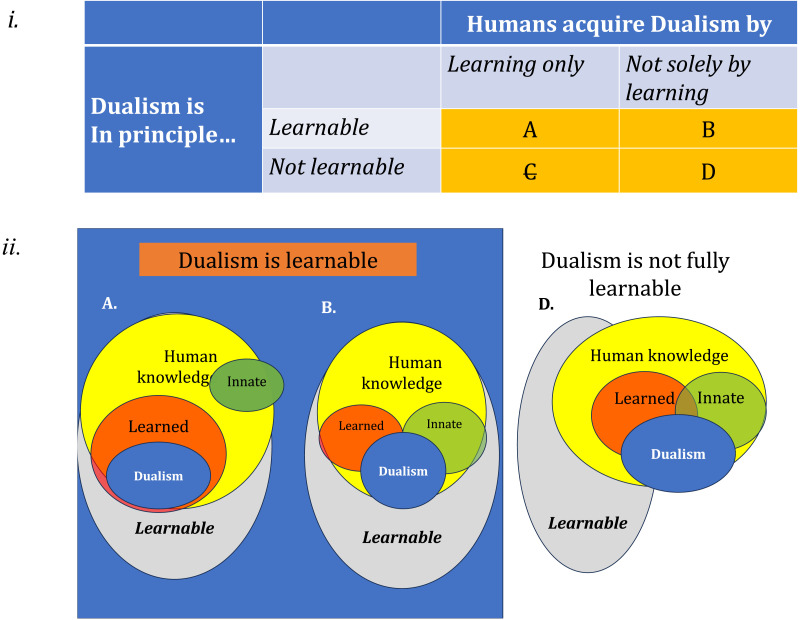
The learnability of Dualism and its learning by humans.

The top panel of Figure 1 (i) defines the four logical options; the bottom panel (ii) further zooms in on the intersection between what is learnable and what humans can learn; of interest here is what knowledge guides the acquisition of Dualism by humans—do humans rely on knowledge that is innate or fully learned.

One possibility (option A) is that Dualism is, in principle, learnable from evidence, and that humans acquire Dualism only by learning; if so, innate constraints ought to play no role in the acquisition of Dualism by humans. To capture that option, diagram iiA shows that Dualism forms a subset of learned human knowledge, and it does not intersect with any innate knowledge. Human Dualism, in this view, is fully learned.

Another possibility is that Dualism is learnable, but human learners do not acquire Dualism solely by learning. Rather, human Dualism arises by the conjunction of innate constraints and learning (option B; see also iiB).

Either way, Options A–B predict that Dualism is learnable. So, if either option is true, and if a LLM is further an adequate learner, then per Options A–B, a LLM ought to exhibit evidence for Dualism.

Conversely, Dualism may not be fully learnable from experience; this is shown by options C and D (Panel i). Option C, however, generates a false prediction. This is so because, per Option C, humans can only acquire it by learning, yet Dualism is unlearnable. Option C thus falsely predicts that humans cannot acquire Dualism—contrary to the facts. Option C, then, is ruled out.

Nonetheless, even if Dualism were not fully learnable from experience, human learners could still acquire Dualism if they were guided by the conjunction of learning mechanisms and innate knowledge (per Option D). Option D, then, generates a clear testable prediction: a LLM (which is devoid of innate knowledge) will fail to fully acquire Dualism, whereas humans will succeed.

Testing the learnability of Dualism by a LLM can thus help adjudicate between these predictions. As noted, the implications to humans are indirect. In particular, null results (failure to learn Dualism) may not be informative, as the failure of a particular LLM may be due to idiosyncratic limitations, and as such, it cannot establish that Dualism is unlearnable. A positive result (i.e., learning success), however, would show that Dualism is learnable—in line with Options A–B above. While this finding could not decide between these two options, it would speak against the strong possibility that Dualism is unlearnable, and thus, can only emerge by innate knowledge (option D). Accordingly, in this research, we seek to evaluate the principled learnability of intuitive Dualism.

## PROBING THE LEARNABILITY OF DUALISM

The following studies ask two questions: (a) Does Davinci show evidence for Dualism; and (b) Do these Dualist tendencies increase as the model’s inductive potential improves?

### Davinci’s Dualist Intuitions

If Dualism is learnable (and if our experimental manipulations adequately gauge Dualism), then like humans, Davinci ought to “consider” the mind as ethereal, distinct from the body. To probe Dualism, we presented Davinci with various psychological traits, and gauged its “intuitions” regarding their anchoring in the body, using the methods of past human research and similar materials (Berent, 2023c; Berent et al., 2019, 2021, 2022). We evaluated Dualism along two distinct approaches.

The first approach evaluates Davinci’s “intuitions” about the propensity of psychological traits to “show up” in a human brain—that is, in the body. If Davinci considers psychological traits as part of the brain (i.e., body), then it ought to view them as likely to manifest in a brain scan.

Past research has shown that humans do not consider all psychological traits as equally likely to manifest in the brain. In particular, people systematically view thoughts (e.g., having a notion of a person; hereafter: *epistemic* traits) as less likely to manifest in the brain than other psychological characteristics, such as sensations, motor plans and emotions—collectively, *non-epistemic* traits (Berent, 2023a, 2023b, 2023c; Berent & Platt, 2021; Berent et al., 2021, 2022).

The human intuition that epistemic traits are less likely to “show up” in the brain is not due to some peculiarity of brain imaging. Indeed, these results closely agree with other probes of embodiment, including tasks that gauge the propensity of psychological traits to transfer to a replica of a person’s body (Berent et al., 2021) and to manifest in specific body organs (e.g., in the face or gut; Berent et al., 2020). Together, these results suggest that the “in the brain” probe indeed gauges the perceived embodiment of psychological traits, and that people consider epistemic traits as particularly disembodied. Of interest is whether Davinci would exhibit similar intuitions.

To find out, Studies 1–2 present Davinci with psychological traits of two kinds. Half of the traits were epistemic (e.g., being able to tell right from wrong); the other half were *non-epistemic* traits—emotions (e.g., love) and actions (squatting down). If Davinci veers towards Dualism, then like humans, it ought to consider epistemic traits as less likely to “show up” in the brain.

It is possible, however, that Davinci’s “in the brain” responses arise for reasons that are unrelated to Dualism (e.g., a bias towards a “yes” response). To evaluate this possibility, Study 3 examines the converse of embodiment. Here, Davinci is asked whether the same traits would emerge in the afterlife, after the body’s demise (i.e., disembodiment).

Past research has shown that when probed about the afterlife, humans consider thoughts as *more* likely to emerge in the afterlife (compared to other psychological states—motor plans and emotions; Berent, 2023c; Berent et al., 2022); this is just the opposite of their response in the “in the brain” scenario (where epistemic traits seem *less* likely to emerge). Thus, human responses systematically *shift* depending on the scenario—whether it targets the body or its demise. If GPT responses likewise reflect Dualism, then its responses should likely change when the probe changes from “in the brain” to “afterlife” scenario.

### Version Comparison

In addition to exploring whether GPT shows evidence for Dualism, our studies further sought to evaluate whether the strength of these Dualist tendencies increase from one model to the next.

To this end, Studies 1–2 probed for the embodiment (“in the brain”) bias across two different versions: Davinci—which forms part of the GPT 3 suite, and its descendent, text-davinci-003—a GPT 3.5 model. Hereafter, we refer to these two versions as *GPT 3* and *GPT 3.5*, respectively (because the log probability function is not available in GPT 4, we did not further evaluate that model).

If Dualism is learnable, then as the LLM’s inductive potential increases (from GPT 3 to GPT 3.5), so should its Dualism and its resemblance to human behavior.

## STUDY 1: “IN THE BRAIN” PROBE (*GPT-3*)

Study 1 asked *GPT 3* to predict the propensity of 80 psychological traits to show up in a brain scan. Half of the traits were epistemic (thoughts; e.g., contrasting right and wrong), and half non-epistemic (motor and affective; e.g., squatting down; anger).

All traits were extensively used in past research in humans (Berent, 2023c; Berent et al., 2019, 2021, 2022). Instructions to humans, however, introduced the brain scan scenario once (at the beginning of the experiment), whereas for GPT, this method of probing was not feasible, as each trait was presented as a separate Application Programming Interface (API) probe, and therefore, each such probe had no memory of previous trials (OpenAI, n.d.-a; Schade, 2023); this was done in order to ensure the independence of the trials from each other.

To direct GPT to judge the trait in the brain-scan scenario, it was therefore necessary to explicitly introduce the “brain scan” scenario within each trial. Accordingly, each trial included a preamble that explicitly references the brain-scan scenario. To minimize the possibility that GPT could accept/reject certain traits simply because it “believes” that these traits are likely/unlikely to exist (as opposed to likely to “show up” in the brain, specifically), each prompt further affirmed that a protagonist (John) holds that trait (e.g., John can distinguish right and wrong). Thus, in the critical experimental trials, each trial featured the trait, the preamble, and the brain-scan query.

Trial order was randomized, and the “temperature” parameter was set to 0 (to minimize variance in the model’s response). In doing so, we sought to increase the consistency of response without making the model fully deterministic (OpenAI, n.d.-b). The “logprobs” parameter was set to 10 (to return the 10 most probable responses with their corresponding probabilities; for the full code, see Appendix I).

### Results and Discussion

Results capture the proportion of “yes” responses relative to the total “yes” and “no” responses. All analyses were conducted using items as random variable.

Extensive pilot work, modeled after the wording of the experimental probes, confirmed that Davinci can appropriately discriminate between questions requiring “yes” vs. “no” responses, and that GPT 3.5 performance indeed exceeded that of GPT 3 (see SM, and Appendix III). Still, past research has shown that the performance of large language models is highly sensitive to specific wording (Mitchell & Krakauer, 2023). Accordingly, we systematically compared responses across multiple versions.

**Figure 2.**
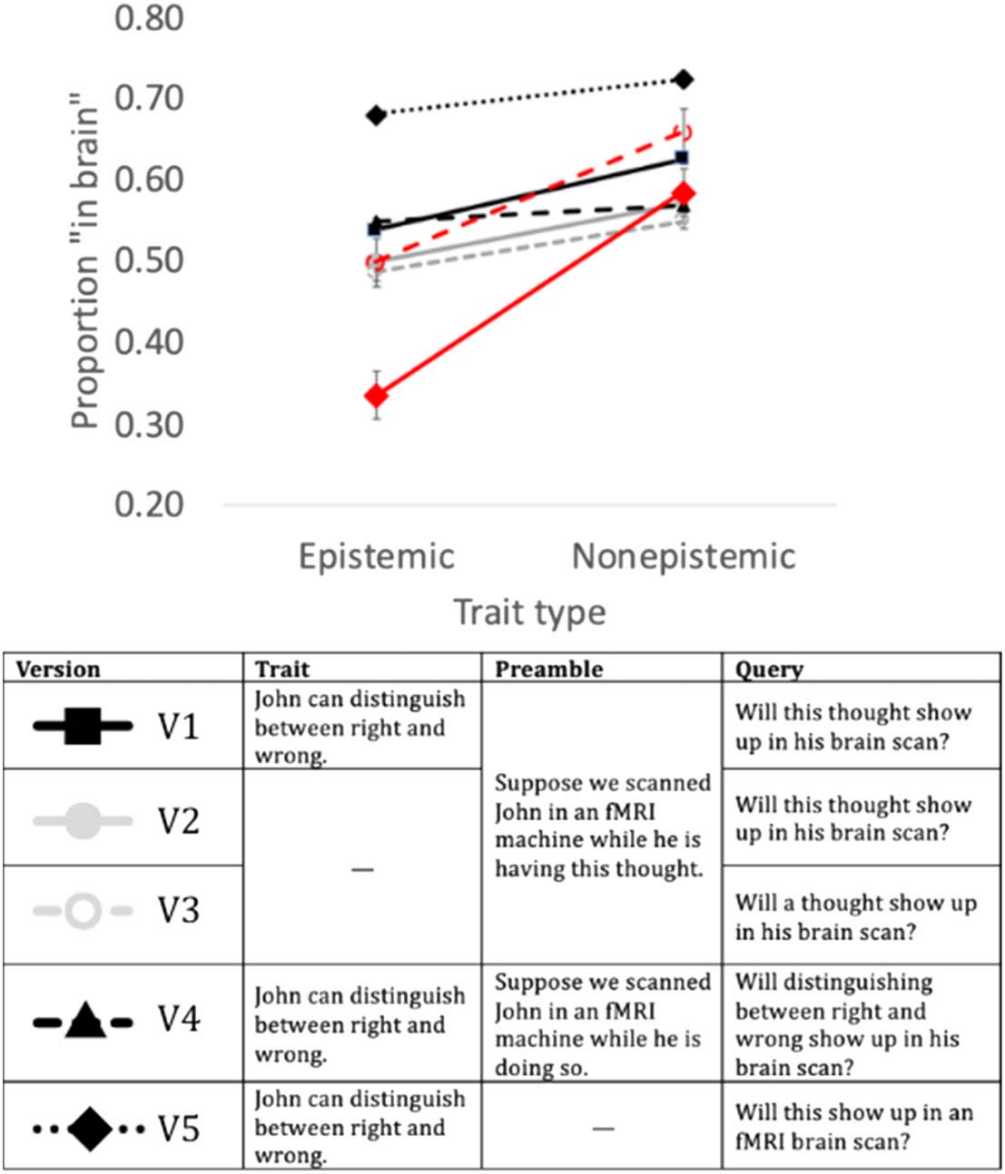
The effect of trait type on *GPT-3* and humans. *Note*: The red lines reflect human responses; the discontinuous line is data from past research using the same traits (Berent et al., 2022); the continuous line captures human response to V5. Error bars are *SE*.

Altogether, we tested GPT on five conditions (Figure 2): three “experimental” conditions probed GPT’s response to individual psychological traits (results are depicted in black); in the two control conditions, the trait was omitted (in gray).

For comparison, we also provide results from two comparable human experiments (results are in red): one set of data captured results to the same traits from past published research (Berent et al., 2022); a second experiment presented humans with the exact wording presented to GPT (of Version 5).

#### Results at a Glance.

An inspection of the means suggests that, across versions, GPT-3 was somewhat less likely to provide a “yes” response for epistemic traits (i.e., thoughts) relative to non-epistemic states—this is in line with past research with humans, who consider epistemic traits less strongly embodied than non-epistemic traits (e.g., actions and emotions).

Still, GPT-3 seems far less sensitive to trait type than humans (in red). Moreover, while humans considered epistemic traits as unlikely to show up in the brain (i.e., below chance), this was never the case for GPT-3. We next detail the results for each version separately and evaluate the reliability of these trends.

#### Detailed Results.

GPT was tested in five different versions (three experimental conditions and two control conditions). The first experimental version presented to *GPT 3* (Version 1) included three sentences. The first affirmed that an individual, John, exhibits the psychological state in question (e.g., *John can distinguish between right and wrong*). The second sentence stated that John underwent an fMRI scan while he is experiencing that state (e.g., a thought). The third sentence (the query) asked whether that state will show up in John’s brain scan. In this and all subsequent studies, all queries concluded with the instruction to respond using yes/no only (for the full text and data, see Appendix II).

Like humans, *GPT 3* considered epistemic traits less likely to manifest in the brain, and this was confirmed by a two-sample t-test (for statistical results, see Table 1). However, the difference between mean response to epistemic and non-epistemic traits was smaller in *GPT 3* (Δ = 0.09) than in humans in our past research (Δ = 0.16). Furthermore, unlike humans, *GPT 3*’s responses to epistemic traits were firmly above chance (Table 1B).

**Table 1.** Statistical results for Study 1.

(A) *The effect of trait type*						
**Version**		**ΔTrait**		***t*(78)**	***p***	**Cohen’s *d***
1		0.09		−8.70	< .001	−1.946
2		0.07		−23.40	< .001	−5.233
3		0.06		−8.18	< .001	−1.829
4		0.02		−1.54	0.127	−0.345
5		0.04		−2.83	0.006	−0.634
Human (published)		0.16		−3.82	< .001	−0.854
Humans (V5)		0.25		−5.85	< .001	−1.308

(B) *Contrasts against chance*						
	**Version**	**Mean**	***SE***	***t*(39)**	***p***	**Cohen’s *d***
Non-epistemic	1	0.63	0.05	17.38	< .001	13.78
	2	0.57	0.02	23.43	< .001	29.73
	3	0.55	0.05	6.38	< .001	11.39
	4	0.57	0.05	8.79	< .001	11.60
	5	0.72	0.05	31.39	< .001	16.10
	Human (published)	0.66	0.17	5.95	< .001	3.93
	Humans (V5)	0.58	0.15	3.51	0.001	3.87
Epistemic	1	0.54	0.04	5.47	< .001	12.29
	2	0.50	0.00	−1.79	0.082	156.17
	3	0.49	0.00	−27.92	< .001	154.91
	4	0.55	0.06	4.89	< .001	8.73
	5	0.68	0.08	13.70	< .001	8.17
	Human (published)	0.50	0.20	−0.05	0.959	2.45
	Humans (V5)	0.34	0.22	−4.68	< .001	1.50

It is possible, however, that *GPT 3*’s response was driven not by the individual trait (e.g., “distinguishing between right and wrong”) but by the trait category (e.g., “thought”), stated in the query. To determine whether the category is *sufficient* to explain *GPT 3*’s responses, we ran two control conditions (Versions 2–3; Figure 2 marks them in gray), featuring only the trait instance (*without* the category). Thus, Versions 2–3 both eliminated the first sentence (introducing the trait instance); Version 2 referenced the trait category as “this” (e.g., *this* thought); Version 3 used “a” (e.g., *a* thought; see Figure 2).

Responses to the two control conditions were similar, and in both, the overall proportion of “yes” responses decreased (relative to Version 1). Still, each version produced a significant effect of trait type, albeit smaller than in humans. Thus, Versions 2–3 demonstrate that category information (e.g., “thought”) is sufficient to elicit the effect of trait type, even when the specific trait (e.g., distinguishing between right and wrong) is removed. The results of these control conditions suggest that *GPT 3* associates thoughts with the brain less strongly than non-epistemic traits (emotions, actions).

Two additional experimental conditions (Versions 4–5) examined the converse—whether *GPT 3* would remain sensitive to trait type when presented the trait instance alone (without the category). Both versions were modeled after Version 1, but the category information was removed, and replaced by either a restatement of the trait instance (e.g., *Will distinguishing between right and wrong show up in his brain scan?* in Version 4) or with “this” (e.g., *Will this show up in an fMRI brain scan?* in Version 5).

Once the trait category was removed, the effect of trait was drastically attenuated (Δ = 0.02 and Δ = 0.04 in Versions 4–5, respectively), in Version 4, it was no longer significant and in Version 5 (with *this*), a strong “yes” bias emerged. Still, responses to epistemic traits remained well above chance, suggesting that, unlike humans, *GPT 3* considered thoughts as likely to show up in the brain.

#### Contrasting *GPT-3* and Humans.

The results discussed thus far suggest that *GPT-3*’s performance was far less sensitive to trait type than humans in our past research. One concern, however, is that the wording of the traits in our past research did not exactly match the one presented to *GPT-3*. To directly contrast *GPT 3* with humans, we next presented the precise wording of Version 5 to a new group of 20 human participants (Prolific workers).

**Figure 3.**
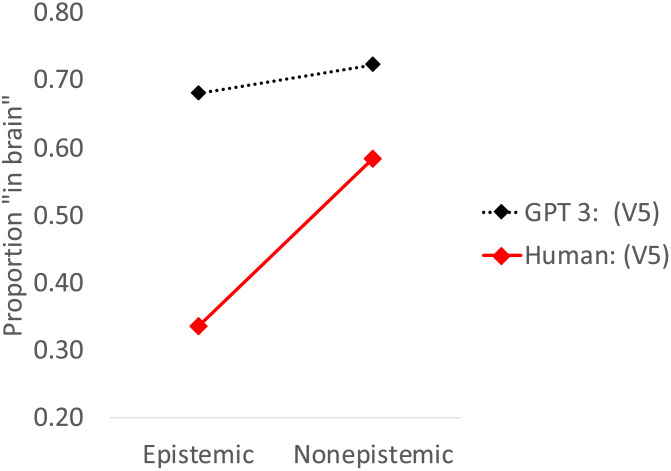
*GPT 3* and human response to Version 5. Error bars are *SE*.

Results (Figure 3) showed that both learners—human and *GPT 3*—considered epistemic traits as significantly less likely to show up in the brain (Table 1), and their responses significantly correlated (*r*(78) = 0.296, *p* = .008). Nonetheless, the effect of trait was six-fold larger in humans (Δ = 0.25) than in *GPT 3* (Δ = 0.04).

Accordingly, a 2 Learner (Human/GPT 3) × 2 Trait (Epistemic/Non-epistemic) ANOVA yielded a reliable interaction (*F*(1, 78) = 23.27, *p* < .001, *η*_p_^2^ = 0.23). Moreover, unlike *GPT 3*, humans now outright denied that epistemic traits will show up in the brain (i.e., responses were significantly below chance).

Summarizing, Study 1 showed that, like humans, *GPT 3* considers thoughts as less likely to “show up” in the brain, especially when given the trait category (e.g., “thought”). However, the effect of trait type was far weaker in *GPT 3*, as most responses hovered close to chance (with the exception of Version 5), and unlike humans, *GPT 3* never denied that thoughts would “show up” in the brain (below chance). Still, the results suggest that the mind–body divide is learnable from human narratives. If so, then the more powerful *GPT 3.5* (i.e., text-*GPT 3.5*) ought to show a stronger Dualist bias. Study 2 evaluates this possibility.

## STUDY 2: “IN THE BRAIN” PROBE (*GPT-3.5*)

Study 2 presented the “in the brain” probe to *GPT 3.5*, following the same procedures advanced in Study 1, and compared it to the human response (to Version 5).

### Results at a Glance

**Figure 4.**
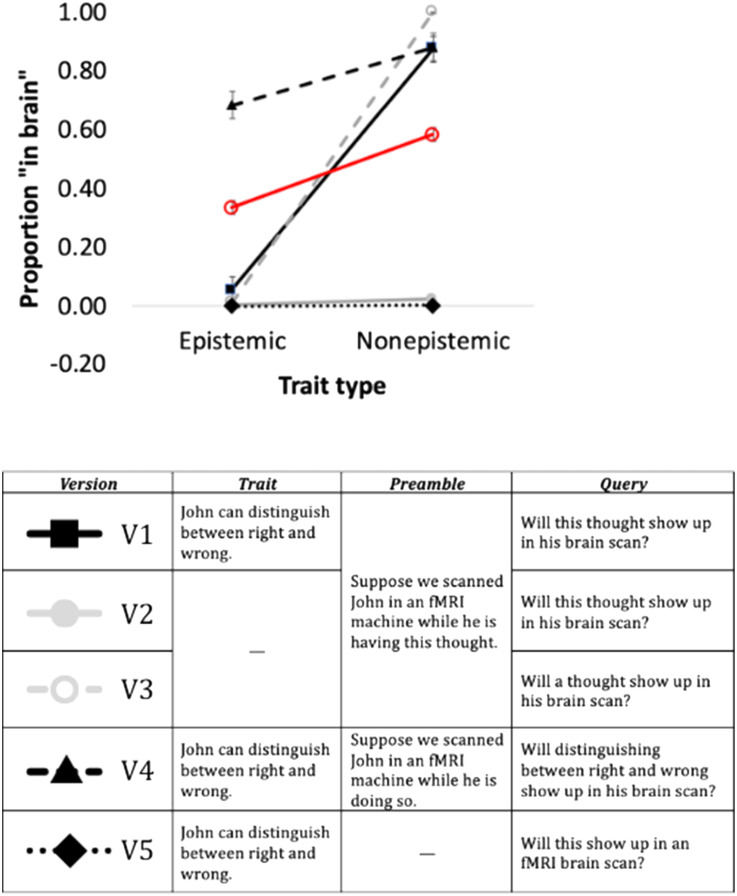
The effect of trait type on *GPT 3.5* and humans. Error bars are *SE*. *Note*: The red line capture human response to V5.

An inspection of the means (Figure 4) suggests that most versions of *GPT 3.5* showed a far stronger effect of trait type (relative to *GPT-3*) that was more akin to humans. As with *GPT-3*, however, the response of *GPT-3.5* varied by the wording of the probe, so we now move to inspect the results across versions.

### Detailed Results

When presented with both the Trait and Category (in Version 1), *GPT 3.5* showed a robust effect of trait type (Figure 4), and the difference between the means (Δ = 0.82) was far larger than in humans (Δ = 0.25). Moreover, like humans (and unlike *GPT 3*), *GPT 3.5* further denied that epistemic traits would emerge in the brain, as its mean response to epistemic traits was significantly below chance (Table 2).

**Table 2.** Statistical results for Study 2.

(A) *The effect of trait type*							
**Version**				**ΔTrait**	***t*(78)**	***p***	**Cohen’s *d***
V1		Category + Trait		0.82	−16.87	< .001	−3.77
V2		Category (this)		0.02	NA		
V3		Category (a)		0.99	NA		
V4		Trait (detailed)		0.20	−2.37	0.02	−0.53
V5		Trait (brief)		0.00	NA	0.061	−0.42
Human		Brief (V5)		0.25	−5.85	< .001	−1.31

(B) *Contrasts against chance*							
		**Version**	**Mean**	***SE***	***t*(39)**	***p***	**Cohen’s *d***
Non-epistemic	V1	Category + Trait	0.88	0.04	8.62	< .001	1.36
	V2	Category (this)	0.02	0.00	NA		
	V3	Category (a)	1.00	0.00	NA		
	V4	Trait (detailed)	0.88	0.05	8.15	< .001	1.29
	V5	Trait (brief)	0.00	0.00	NA		
	Human	Brief (V5)	0.58	0.02	3.51	0.001	0.56
Epistemic	V1	Category + Trait	0.05	0.02	−20.46	< .001	−3.24
	V2	Category (this)	0.00	0.00	NA		
	V3	Category (a)	0.01	0.00	NA		
	V4	Trait (detailed)	0.68	0.07	2.64	0.012	0.42
	V5	Trait (brief)	0.00	0.00	NA		
	Human	Brief (V5)	0.34	0.04	−4.68	< .001	−0.74

This large effect, however, was primarily due to the explicit labeling of the trait category (e.g., “thought”). Indeed, it persisted even when the specific trait instance was removed (in the Control Versions 2–3). Like *GPT 3*, however, *GPT 3.5* appears to struggle with the syntactic determiner “this”. Thus, when the Category was referenced by “this” (In Version 2), *GPT 3.5* invariably responded “no”, whereas its responses to “a” (In Version 3) yielded nearly uniform “yes” response to non-epistemic traits, and “no”, to epistemic traits.

**Figure 5.**
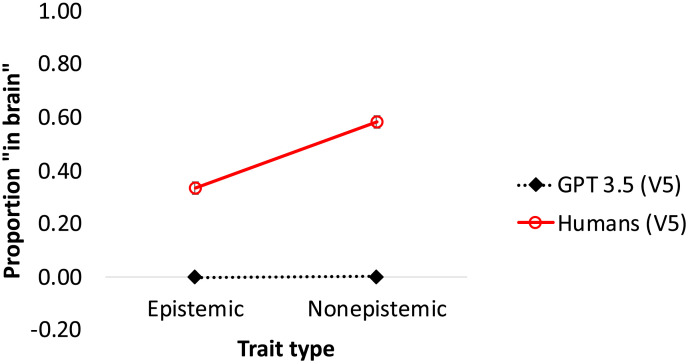
*GPT 3.5* and human response to Version 5. Error bars are *SE*.

Still, when *GPT 3.5* was given *only* the trait instance (without any category information, in Versions 4), it indicated that epistemic traits *would* show up in the brain, less so than non-epistemic traits. This effect, however, only obtained when the trait was explicitly referenced in the query (e.g., *will distinguishing right from wrong show up in the brain*, in Version 4). When the trait was referenced by “this” (Version 5), response was once again at floor. This clearly differs from human response to Version 5 (see Figure 5).

Since *GPT 3.5*’s response to Version 5 had no variance, to examine the correlation with human learners, we compared human behavior with *GPT 3.5*’s response to Version 4 (which likewise featured only the trait instance); the correlation was significant (*r*(78) = 0.468, *p* < .001).

Summarizing, *GPT 3.5*’s responses were highly dependent on the specific wording. Moreover, unlike humans, when *GPT 3.5* was only presented with the trait information (in Versions 4–5), *GPT 3.5* did not ***selectively*** indicate that epistemic traits *won’t* show up in the brain (i.e., below chance). In particular, Version 4 stated that epistemic states *will* emerge in the brain, whereas Version 5 was at floor across the board, for *both* epistemic and non-epistemic traits (i.e., it showed no *selectivity*). Still, compared to *GPT 3*, *GPT 3.5* showed a far stronger sensitivity to trait type, and its responses correlated with and human behavior.

## STUDY 3: AFTERLIFE PROBE (*GPT 3.5*)

The results Study 2, then, are in line with the possibility that *GPT 3.5* has acquired a Dualist bias. It is possible, however, that its lower “yes” response to epistemic traits arises for some other reasons (e.g., a bias to respond “yes”).

The hallmark of Dualism, however, is selectivity: while humans consider thoughts as less likely to show up in the brain, they typically consider them *more* likely to emerge in the afterlife—after the body’s demise (e.g., Berent, 2023c; Berent et al., 2022). To determine whether *GPT 3.5* indeed leans towards Dualism, in Study 3, we thus examined whether the bias against epistemic traits would change—either eliminated, or possibly, reversed—when *GPT 3.5* is asked about the persistence of these traits after a person dies.

**Figure 6.**
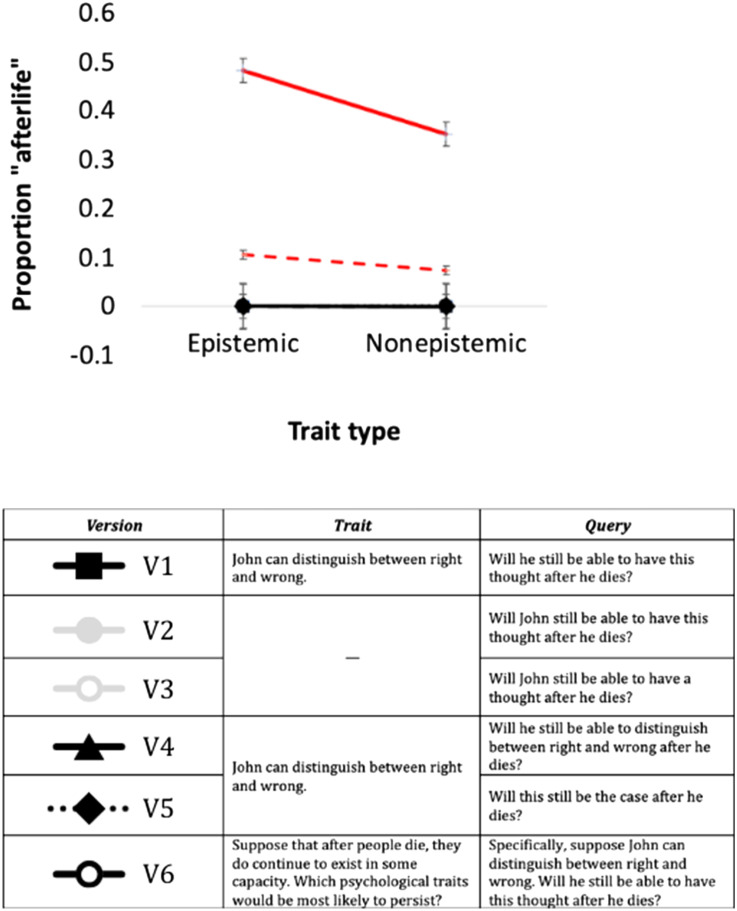
The effect of trait type on *GPT 3.5* and humans’ responses to the afterlife probe. Error bars are *SE*. *Note*: Red lines depict human response to V5 when the afterlife is either assumed to exist (continuous line) or no such assumption is made (discontinuous line).

The probe structure followed that of the brain queries in Studies 1–2. In Version 1, the first sentence introduced the trait (e.g., *John can distinguish between right and wrong*); the second introduced the query (*Will he still be able to experience this emotion after he dies?*). Versions 2–3 (Controls) presented the query only; Versions 4–5 removed the category information (see Figure 6).

An inspection of the means suggests that *GPT 3.5* categorically denied the afterlife—its response in ***all*** versions was uniformly “no” (*M* = 0.00; *SD* = 0.00). This phenomenon, where *GPT 3.5* treats an open-ended question categorically, as if there was a pre-set “correct” answer, agrees with past research (Park et al., 2024).

This so-called “correct effect” in GPT contrasts with neurotypical participants in our past research, who stated that epistemic traits are not only *more* likely to persist (relative to non-epistemic traits) but also insisted that they *will* persist—above chance (Berent, 2023c; Berent et al., 2022). This was also the case when human participants (Prolific workers, *N* = 20) were presented with the precise wording of Version 5 (Δ = 0.13; *t*(78) = 4.94; *p* < .001; Cohen *d* = 0.781; Figure 6 depicts these data in the red continuous line).

Still, the instructions to humans and machines differed. In this and our previous research, humans were instructed to assume that the afterlife exists (hence, in Figure 6, their data is labeled as “afterlife assumed”; these data are depicted by the red discontinuous line), whereas *GPT 3.5* was not told so. Could this explain the difference?

To find out, Version 6 provided *GPT 3.5* with the same instructions (using Version 1, as this version showed a large effect of Trait type, in Study 2). We also performed the converse on humans (*N* = 20, Prolific workers)—we asked them to simply indicate whether after John dies, he would still be able to show the same abilities he had in life.

Results were clearcut: When the afterlife was assumed, *GPT 3.5*’s responses remained “no” (Mean = 0.00, *SD* = 0.00). Humans, by contrast, showed a small, but significant tendency to consider epistemic traits more likely to emerge, even when the afterlife was no longer assumed (Δ = 0.032, *t*(19) = 2.43, *p* = .017, Cohen *d* = 0.544). Still, the instructions mattered, as now, responses approached floor, and epistemic traits were not endorsed (above chance).

Summarizing, Study 3 confirms that, when probed about the afterlife, *GPT 3.5* no longer considers epistemic traits as less likely to emerge (unlike its response to the brain probe, in Study 2); this is in line with Dualism, and it also agrees with human behavior. Unlike humans, however, *GPT 3.5* never considered epistemic traits *more* likely to persist after death; indeed, it singlehandedly denied that the psyche persists after death.

Thus, the response of *GPT 3.5 * was selective with respect to the scenario (brain vs. afterlife), but in the afterlife scenario, it was non-selective with respect to trait type. We next turn to interpret this result in the context of the findings of Studies 1–3 as a whole.

## GENERAL DISCUSSION

Humans are intuitive Dualists—they tend to view the mind as ethereal, distinct from the body. Here, we asked whether the experience available to humans is in principle *sufficient* to elicit the Dualist bias.

To address the contribution of learning, we contrasted Dualist intuitions in humans with Davinci, an LLM. We reasoned that, if the Dualist bias is learnable from experience (and our experimental manipulations adequately gauge Dualism), and if Davinci can adequately capture human learning, then Davinci’s intuitions about bodies and minds ought to resemble humans’. Furthermore, as Davinci’s inductive capacities improve—from its earlier version to a later one, Dualist tendencies ought to increase.

In line with this prediction, Study 1 showed a mild Dualist bias in davinci (a member of the GPT 3 suite, hereafter, *GPT 3*), as it “considered” epistemic traits as somewhat less likely to manifest in the brain than non-epistemic traits—in line with human behavior.

Text-davinci-003 (from the GPT 3.5 suite, hereafter, *GPT 3.5*) showed a far stronger sensitivity to trait type (in Study 2). Not only did *GPT 3.5* “consider” thoughts (i.e., epistemic traits) *less* like to show up in the brain, but in fact, it categorically “asserted” that they won’t (i.e., it responded below chance), and its behavior correlated with humans.

To demonstrate that the bias against epistemic states specifically concerns their propensity to emerge in the body, Study 3 next probed *GPT 3.5*’s intuitions regarding the potential of the same traits to persist after the body’s demise—in the afterlife.

As expected, here, *GPT 3.5* no longer considered epistemic traits as less likely to emerge. Instead, it singlehandedly denied that life can persist after death, and treated this belief as a matter of fact (Park et al., 2024).

In light of Study 3’s findings, one might be tempted to conclude that *GPT 3.5* is a staunch Physicalist, as it denies that the mind can outlive the body. This interpretation, however, fails to explain why *GPT 3.5* considered epistemic states as less likely to show up in the brain—precisely as Dualism predicts.

To account for the entire pattern of findings, here, we suggest that *GPT 3.5* has acquired *two* conflicting biases. On the one hand, GPT is biased towards Dualism. But in tandem, it has also induced a separate belief that life ceases at death. The tension between this seemingly Physicalist belief and Dualism is also evident in the human participants in Study 3. At no point did these participants explicitly endorse the afterlife (above chance)—in line with Physicalism. And yet, they still considered epistemic states more ethereal (i.e., as likely to emerge in the afterlife, but not in the brain), in line with Dualism.

Similar conflicting biases are also reported in past research. For example, Jesse Bering (Bering, 2002) observed that, when participants are explicitly probed for their afterlife beliefs, some individuals claim that the self is extinguished at death. But when probed implicitly, these same “extinctivists” still considered epistemic states as more likely to persist after death (more so than biological traits), in line with Dualism.

Thus, Physicalist/extinctivist and Dualist beliefs can well co-exist side by side. Humans, for instance, can learn that thinking “happens” in the brain and ceases after death (e.g., through their science training), but at heart, remain “closet Dualists. This tension is only expected by the hypothesis that intuitive Dualism is a “soft”, violable bias, rather than an absolute inviolable rule (Berent, 2023b). The mixed response of *GPT 3.5* appears to reflect the same tension.

Altogether, then, the results yield mixed evidence for Dualism. While, in the afterlife condition, GPT 3.5 denies that the mind can outlive the body, when asked about the propensity of the psyche to “show up” in the brain, here, it seems to consider epistemic traits as disembodied (i.e., as less likely to “show up” in the brain), just as humans do.

To our knowledge, this is the first study to document a Dualist bias in AI. To be sure, these results do not imply that GPT holds any beliefs about bodies and minds, or any other type of mental states. Rather, the finding suggests that GPT can induce a Dualist bias from the human texts available to it. These results also converge with past research, showing that as AI is exposed to human narratives, it acquires not only human knowledge but also human biases (e.g., Bordia & Bowman, 2019).

That such a bias has emerged is far from trivial, as responses to bodies and minds were not probed explicitly. In particular, we did not ask Davinci whether the mind is distinct from the body—a belief that is extensively expressed in the Western cultural canon, and thus, trivially learnable. Rather, we probed for implicit attitudes about the emergence of numerous, specific psychological traits in manipulations that either target the body (i.e., a brain scan) or its absence (i.e., the afterlife); there were no mentions of body and mind, and in some versions, we did not even mention the trait type (e.g., thought). Remarkably, the results of *GPT 3.5* showed several similarities to humans.

Still, *GPT 3.5*’s behavior differed from humans in several respects. First, as noted, *GPT 3.5* never considered epistemic traits as *more* like to persist in the afterlife (compared to non-epistemic traits), and indeed, it singlehandedly denied that any trait persists after death. Second, when probed about the brain, *GPT 3.5 selectively* rejected the emergence of epistemic traits (i.e., below-chance response to epistemic, but not to non-epistemic traits) only when they were explicitly labeled as thoughts. Third, *GPT 3.5*’s behavior was highly sensitive to the specific wording and it struggled with the resolution of syntactic anaphor (this); this is in keeping with the known syntactic limitations of GPT (Leivada et al., 2023).

One possibility, then, is that the divergence arose because human learning capabilities surpass Davinci’s, possibly because human cognition include algebraic “language of thought” (Fodor, 1975; Quilty-Dunn et al., 2023), and these algebraic capacities may not be fully learnable by AI (cf. Lake & Baroni, 2023; Marcus & Davis, 2019). Alternatively, the differences could have emerged because human Dualist intuitions are amplified by additional psychological biases that are unavailable to Davinci, including ones arising from human core knowledge of objects and theory of mind (Bloom, 2004). This proposal can further explain why it is the case that evidence for Dualism arises cross-culturally, and why it is systematically attenuated in individuals in groups that are somewhat less adept at reading the minds of others—in autistic relative to neurotypicals, and within the neurotypical population, in males relative to females (Berent, 2023c; Berent et al., 2022). In this latter view, it is precisely because humans are natural Dualists that human corpora allow Dualist biases to emerge in AI.

Taken as a whole, these results suggest that the evidence available to humans is sufficient for learning some of their intuitions about bodies and minds (given a learning mechanism such as GPT 3.5). As noted, these conclusions about what is learnable (under certain conditions) say nothing about whether humans do in fact learn Dualism in this fashion. Moreover, human behavior differed from Davinci’s in several respects.

Still, our results show for the first time that Dualism is learnable even in the absence of any innate, domain-specific biases. This conclusion puts a higher burden of proof on nativist theories that trace Dualism to innate core cognition (Berent, 2023a; Berent et al., 2022).

## AUTHOR CONTRIBUTIONS

Iris Berent: Conceptualization; Formal Analysis; Methodology; Visualization; Writing – original draft. Alexzander Sansiveri: Data Curation; Methodology, Project Administration; Software; Visualization; Writing – review & editing.

## DATA AVAILABILITY

All data are provided in Appendix II.

## Supplementary Material










