# Supplementary material for: Davinci the Dualist: The Mind–Body Divide in Large Language Models and in Human Learners
Source: Open Mind (Camb). 2024 Mar 1;8:84–101. doi: 10.1162/opmi_a_00120 (PMC10898781; doi:10.1162/opmi_a_00120)
Supplement: Supplementary file 1 [file opmi-08-84-s001.docx]

**Davinci the Dualist:**

**the mind-body divide in large language models and in human learners**

**Supplementary Materials**

Iris Berent

Alexzander Sansiveri

Northeastern University

Address for correspondence:

Iris Berent, Ph.D

Department of Psychology

Northeastern University

125 Huntington Ave.

Boston, MA 02115

[I.berent@northeastern.edu](mailto:I.berent@northeastern.edu)

1. ***Simulation Method***

All simulations followed the same procedure. Each simulation presented Davinci (either GPT 3, in Study 1, or GPT 3.5, in Studies 2-3) with a list of 80 psychological traits, using five distinct versions, as detailed in the Main Text.

Each trait was presented as a separate, individual query (with order randomized); the beginning and end of each such query was marked by “q:” and “a:”, respectively (e.g., “*q: John can feel anger in response to hostility. Will this show up in an fMRI brain scan? You may only answer yes or no. a:”*). The “temperature” parameter was set to 0 (to minimize unnecessary variance in the model’s response), and “logprobs” was set to 10 (to return the 10 most probable responses with their corresponding probabilities).

To calculate the proportion of binary responses, we first edited the output, so responses were invariably presented in lowercase. We then checked to determine the first word was either “yes” or “no”, extracted their probabilities, and computed the probability of “yes” relative to the probability of “yes” and “no” responses (combined). For the complete code, see Appendix I. The complete materials, along with Davinci’s and Human responses are provided in Appendix II; additional instructions (for human participants) are provided in Appendix III.

1. ***Pretesting Davinci***

Prior to the experimental testing (where the correct answer to the probes is often unknown), we first sought to determine whether Davinci can correctly respond to questions for which there is a known answer, and the format of the question is modeled after Versions 1 and 5. In so doing, we wished to ascertain that (a) Davinci can correctly “understand” and respond to our queries; and that (b) the later GPT-3.5 versions indeed exceed the performance of GPT-3. To this end, we probed Davinci on two sets of questions—(i) brain imaging and (ii) simple arithmetic questions (for the full materials and results, see Appendix IV).

*i. Brain imaging.* The brain imaging probe was closely similar to the “in brain” and “afterlife” questions (in Study 1-2 vs. 3), except that here, the brain questions concerned abilities that are well-known to be detectible in human brains.

There were nine such questions—three probed for sensory abilities (face, shape, and color recognition), three concerned motor actions (moving one’s lips, hands, and legs), and three concerned cognitive abilities (recognizing colors, melodies, and English sentences).

Each probe first affirmed that John possesses the capacity in question (e.g., *John can recognize faces*); and the second established that John undergoes imaging while he is either experiencing the psychological state in question (e.g., *Suppose we scanned John in an fMRI machine while he is experiencing this sensation*) or after he dies (e.g., *Suppose we scanned John in an fMRI machine after he dies).* The probe asked whether this capacity would show up in the brain scan. Thus, in the ‘alive’ situation, the correct answer is “yes”; in the death scenario, it’s “no”.

As in the main studies, Version 1 specified the trait category (e.g., *Will this sensation show up in his brain scan?)* whereas Version 5 only uses an anaphor (*Will this show up in an fMRI brain scan?*). Each question concluded with the requirement to respond using only yes/no. Each of the nine questions was repeated 10 times. The full text, along with Davinci’s responses are provided in Appendix IV.


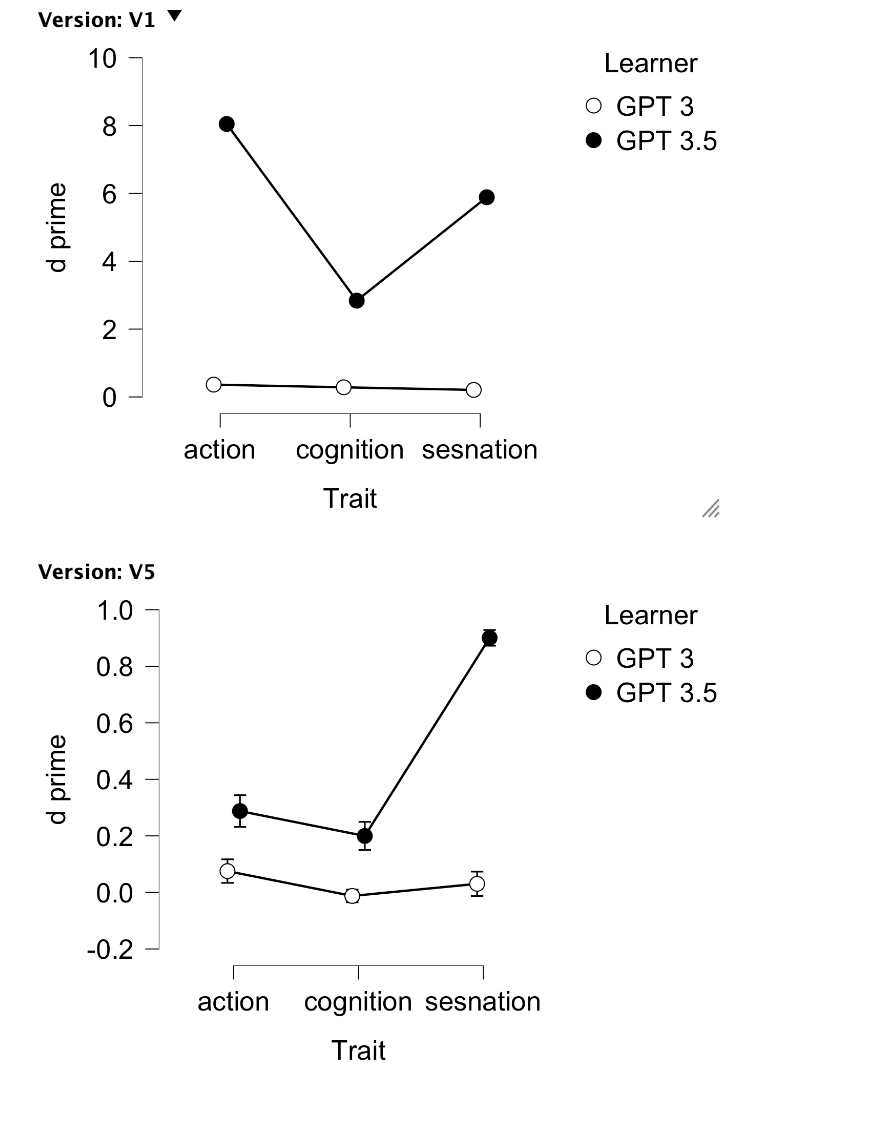

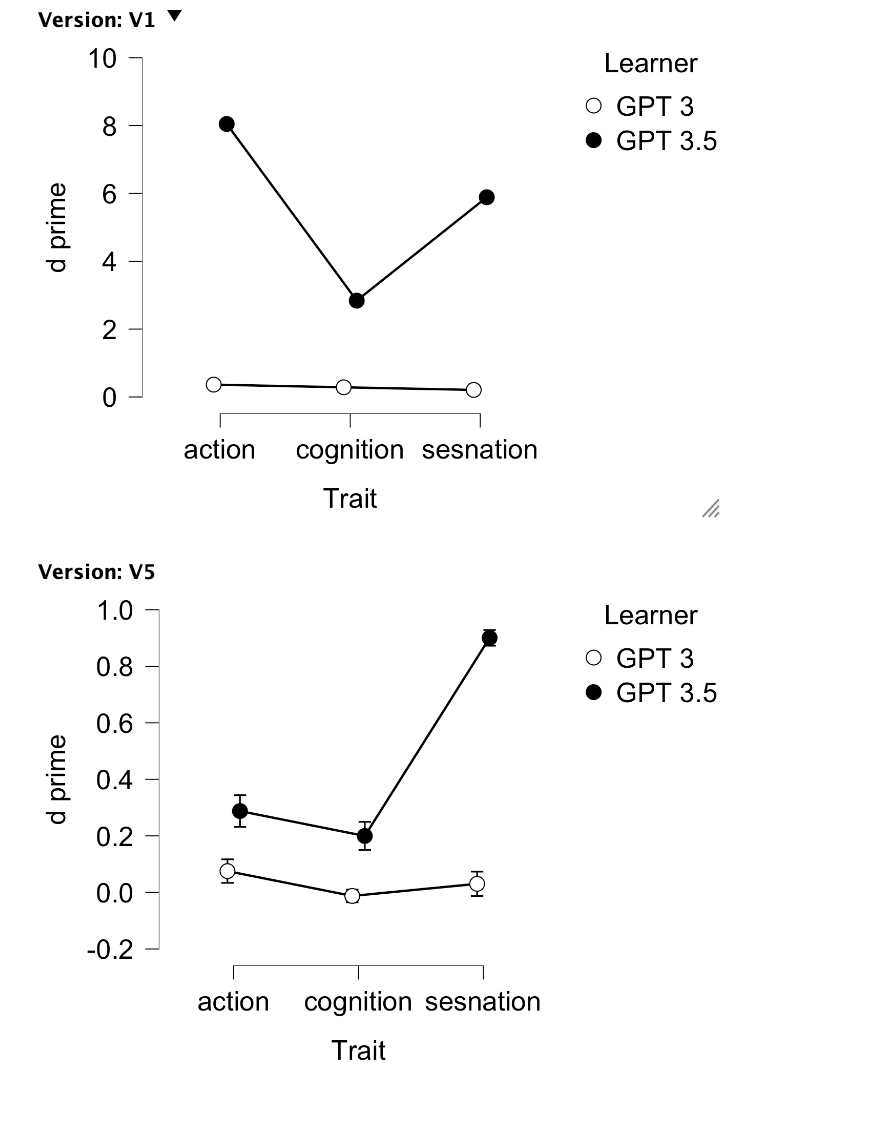


**Figure S1**. The performance of Davinci on the “in brain” practice questions.

Figure S1 provides the mean d prime response (hit=correct “yes” to the “in brain” question). An inspection of the results suggests that, in both versions, GPT 3.5 outperformed GPT 3. Both versions, however, exhibited a strong Dualist bias, as sensitivity to cognitive traits was lower than actions and sensations. This bias, however, was stronger in Version 1 than in Version 5 (with the *this* anaphor). This is in line with the syntactic difficulties of GPT 3.5 with *this*, discussed in the main text.

Accordingly, a 2 Learner (GPT 3 vs. GPT 3.5) x 2 Version (1 vs. 5) x 3 Trait ANOVA yielded a reliable three way interaction (F(2,87)=964.55, p<.001; η² _p_ =0.957). Still, the simple main effect of Leaner was highly significant for each of the traits and versions (all p<.01).

*ii. Arithmetic questions.* A second pretest examined Davinci’s response to simple addition questions, presented using a query structure modeled after Version 1 (e.g., *John can add one plus one. Suppose we had him perform this calculation. Will his answer be two/one? You may only answer yes or no.*)

Here, GPT 3’s mean accuracy was 0.52 whereas GPT 3.5 mean was 1.00. Accordingly, the sensitivity of GPT 3.5 (d’=9.79) clearly exceeded GPT 3 (d’=0.097; F(1, 59)=41,824, p<.001, η² _p_ =0.999).

1. ***Human participants***

Participants in all studies were recruited from Prolific; they were adults, native English speakers, free of any reading-language or neurological disorders.

Sample size matches that in past research using the same procedures (Berent, 2023; Berent, Theodore, & Valencia, 2022), and by power calculations, indicating that the selected sample is sufficient to attain a power of .80 at the alpha level of .05.

The instructions given to participants are provided in Appendix III; all experimental probes and data are presented in Appendix II.

**References**

Berent, I. (2023). The illusion of the mind–body divide is attenuated in males. *Scientific Reports, 13*(1), 6653.

Berent, I., Theodore, R., & Valencia, E. (2022). Autism attenuates the perception of the mind-body divide. *Proceedings of the National Academy of Sciences, 119*(49), e2211628119-e2211628119.
