# Supplementary material for: Davinci the Dualist: The Mind–Body Divide in Large Language Models and in Human Learners
Source: Open Mind (Camb). 2024 Mar 1;8:84–101. doi: 10.1162/opmi_a_00120 (PMC10898781; doi:10.1162/opmi_a_00120)
Supplement: Supplementary file 2 [file opmi-08-84-s002.pdf]

```python
import openai
import pandas as pd
import random

# Set up OpenAI credentials
openai.api_key = 'openai_key HERE'

# Define model ID and other parameters
model_id = 'model_id HERE'
num_of_prompts = 1
kwargs = {"engine":model_id, "temperature":0, "max_tokens":10, "stop":"\n", "logprobs":10}

# Read data from excel file
path = r'path HERE'
df = pd.read_excel(path)

# Shuffle the list of prompt IDs
list_of_ids = list(range(num_of_prompts))
random.shuffle(list_of_ids)

# Loop through the list of prompt IDs
for idx in list_of_ids:
  # Format the prompt
  prompt = "q: " + df["Prompts"][idx] + " \na:"
  print(prompt)

  # Perform the query
  r = openai.Completion.create(prompt=prompt, **kwargs)
  print(r)

  # Set up the dataframe with the results
  scores = pd.DataFrame([r["choices"][0]["logprobs"]["top_logprobs"][0]]).T
  scores.columns = ["logprob"]
  scores["%"] = scores["logprob"].apply(lambda x: 100*np.e**x)

  sumYes = 0
  sumNo = 0

  # Compute the total percentage of 'yes' and 'no' responses
  for index, row in scores.iterrows():
    if index.strip().lower() == "yes":
        sumYes += row['%']
    elif index.strip().lower() == "no":
        sumNo += row['%']

  print(scores)
  print(prompt)
  print("Total percentage of 'yes': ", sumYes)
  print("Total percentage of 'no': ", sumNo)

  # Update the existing data in the Excel file
  df.loc[idx, "prob_Yes"] = sumYes
  df.loc[idx, "prob_No"] = sumNo
  df.loc[idx, "prob_Other"] = 100 - sumYes - sumNo
  df.loc[idx, "yes/yes+no"] = sumYes/(sumYes + sumNo)

# Save the changes to the Excel file
df.to_excel(path)
```
