# Supplementary material for: Davinci the Dualist: The Mind–Body Divide in Large Language Models and in Human Learners
Source: Open Mind (Camb). 2024 Mar 1;8:84–101. doi: 10.1162/opmi_a_00120 (PMC10898781; doi:10.1162/opmi_a_00120)
Supplement: Supplementary file 4 [file opmi-08-84-s004.docx]

**Study 1-2**

**Instructions for the human participants**

|  |
| --- |

In the following experiment, you will be presented with a list of psychological traits. Suppose we were to scan the brain of a person, John, in an fMRI machine while he is experiencing those psychological characteristics. How likely is it that each trait will show up in his brain scan?

Please indicate your answer using a yes/no response (1=yes, this trait will show up in the brain scan; 2=no, this trait will not show up in the brain scan)


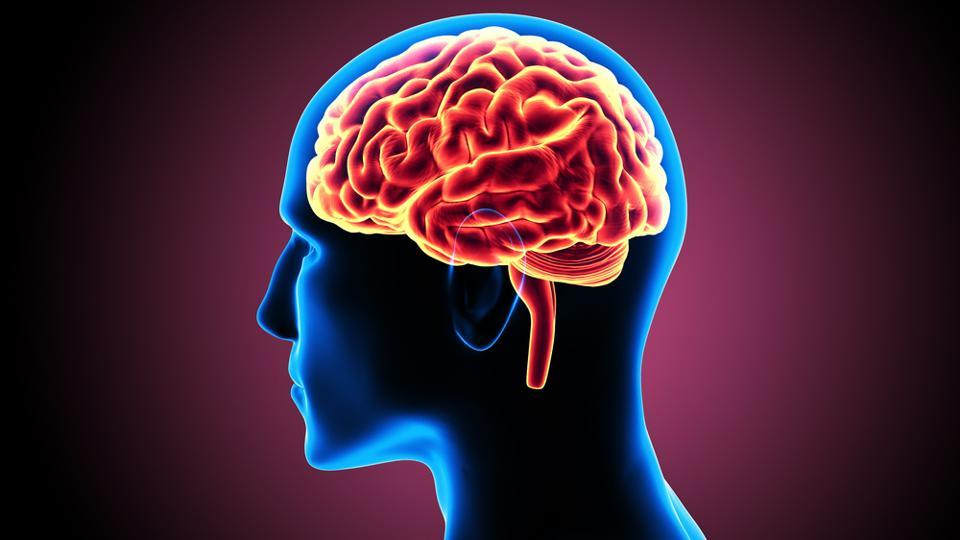


**Study 3**

**Instructions for human participants (afterlife assumed)**

In this experiment, we examine people’s beliefs about the afterlife. Whether or not there is an afterlife, is of course unknown. But for the purpose of this experiment, we invite you to assume that after people die, they do continue to exist in some capacity. Which psychological traits would be most likely to persist?

In the experiment, you will be presented with a list of psychological traits. Suppose a person, John, can experience each of these traits while he is alive. Will this still be the case after he dies?

Please indicate your answer using a yes/no response (1=yes; 2=no)

Thank you!


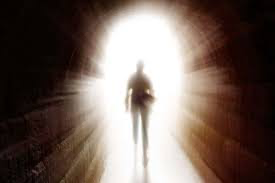


**Study 3**

**Instructions for human participants (afterlife is not assumed)**

In the experiment, you will be presented with a list of psychological traits. Suppose a person, John, can experience each of these traits while he is alive. Will this still be the case after he dies?

Please indicate your answer using a yes/no response (1=yes; 2=no)

Thank you!
